# The neuronal cell cycle as a mechanism of pathogenesis in Alzheimer's disease

**DOI:** 10.18632/aging.100045

**Published:** 2009-04-28

**Authors:** Antonio Currais, Tibor Hortobágyi, Salvador Soriano

**Affiliations:** ^1^Department of Neuroscience, MRC Centre for Neurodegeneration Research, Institute of Psychiatry, King's College London, London SE5 8AF, UK; ^2^Department of Clinical Neuropathology, Academic Neuroscience Centre, King's College Hospital, London, UK and Department of Pathology, University of Szeged, Szeged, Hungary

**Keywords:** cell cycle, neuron, Alzheimer's disease

## Abstract

Differentiated neurons display specific biochemical, physiological and morphological properties that apparently prevent them from further cell division. Nevertheless, expression of cell cycle modulators persists after neuronal differentiation and is upregulated under stress conditions, such as trophic factor deprivation, oxidative stress and the presence of DNA damaging agents. This apparent reactivation of the cell cycle has been postulated as a sine qua non for neuronal death in response to those stress conditions, particularly in Alzheimer's disease. However, the physiological and pathogenic implications of a putative neuronal cell cycle are far from clear. Here, we discuss the notion of the neuronal cell cycle as a mediator of cell death, with particular emphasis on Alzheimer's disease.

## Introduction

**Figure 1.**
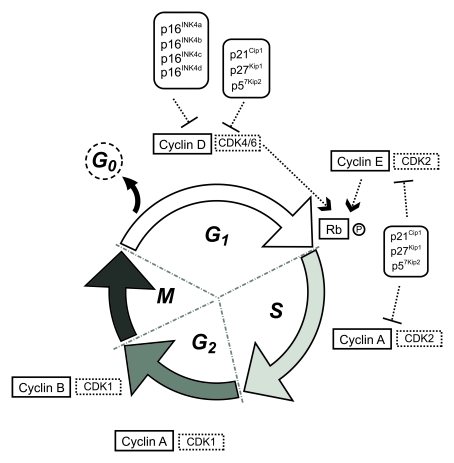
Schematic representation of the eukaryotic cell cycle.

Once a neuron is born, it loses its capacity for cell division and differentiates, contributing uniquely to the plasticity of the basic wiring pattern that defines a neuronal system. The preservation of this pattern is necessary for the overall generation and storage of memories, as well as the acquisition of other higher brain skills. Differentiated neurons appear to be irreversibly post-mitotic, perhaps because a hypothetical cell division would result in cytoskeletal and synaptic disruption in order to prepare the cell for mitosis and cytokinesis, which would in turn impair neuronal connectivity and function. Hence, it is reasonable to hink that, once a neuron differentiates, it resides out of the reach of cell division control. However,  this notion was first questioned when some researchers surprisingly observed that neuronal programmed cell death was accompanied by the expression of cell cycle markers. Specifically, cyclins and cyclin-dependent kinases (CDKs), key components of the cell cycle machinery (see Figure 1) were found upregulated after exposure to severe conditions, such as oxidative stress and trophic factors deprivation [1-10]. Based on the premise that "neurons do not divide", the notion that has emerged from this evidence is that activation of a neuronal cell cycle does exist but it is abortive, the final result being the initiation of apoptosis. As we discuss below, this aberrant phenotype has also been postulated as a mechanism of neuronal loss in neurodegenerative diseases, particularly Alzheimer's disease (AD).

### Regulation of the cell cycle

The cell cycle of eukaryotic cells comprises four main successive phases: G1 phase (first gap), S phase (DNA synthesis), G2 phase (second gap) and M phase (mitosis) (Figure 1). Transition between the different phases and subsequent progression through the mitotic cycle is driven by a group of protein kinases whose activity is central to this process, the cyclin-dependent kinase (CDKs), and requires the binding of their activating partners cyclins, whose levels of expression varies throughout the cycle.

During G1 phase, mitogenic signals, such as extracellular growth factors or intercellular contact, trigger the activation of D-type cyclins that, jointly with CDK4 or CDK6, phosphorylate the retinoblastoma protein (Rb), inhibiting its affinity to bind the transcriptor factor E2F- 1. E2F-1 is released and directs the transcription of specific genes that code for proteins required in the next stages of the cell cycle. In late G1, an increase in cyclin E-CDK2 activity ensures the G1/S transition by completing Rb phosphorylation and irreversibly committing cells to enter the division process. Throughout S phase, cyclin A-CDK2 phosphorylates various substrates allowing DNA replication. After completion of S phase, DNA replication ceases and cells enter the G2 phase of the cycle. CDK2 is then replaced by CDK1 that associates with cyclin A and regulates the phosphorylation of proteins specific to the G2 and M phases of the cell cycle together with cyclin B-CDK1,  that appears in late G2 and triggers the G2/M transition. Cyclin A is degraded and the system is reset, re-establishing the requirement for mitogenic cues to induce D-type cyclins for the next cycle. In M phase, cells physically divide originating two separate daughter cells (reviewed in [11]).

CDK activity is regulated through posttranslational modifications and subcellular translocations of specific CDK inhibitors (CDKIs), which are organized in two families, INK4 and Cip/Kip. The INK4 family (inhibitors of cyclin D-dependent kinases) consists of four members: p16^INK4a^, p15^INK4b^, p18^INK4c^ and p19^INK4d^, and the Cip/Kip family (inhibitors of cyclin D-, cyclin E-, and cyclin A-dependent kinases) comprises p21^Cip1^, p27^Kip1^ and p57^Kip2^.

Two important checkpoints (G1/S and G2/M) coordinate CDKs activity and control the order and timing of cell-cycle transitions ensuring that DNA replication and chromosome segregation are completed correctly before allowing further progress through the cycle. The checkpoints allow alternative decisions between progression, growth arrest or induction of apoptosis. (See [12] for a detailed review addressing the regulation of the cell cycle in proliferating cells).

### Differentiated neurons express cell cycle proteins

Neurogenesis, the birth of differentiated, functional neurons, takes place at two germinal compartments that line the lateral ventricles - the ventricular zone (VZ) and the subventricular zone (SVZ). Most neurons are originated prenatally through a process of migration to shape a complex pattern of layers. The deep layers are formed from earlier-born neurons originated in the VZ, while later-generated neurons from the SVZ occupy higher layers [13]. The journey is meant to cease proliferation and start neuronal differentiation. However, although terminally differentiated neurons seem to irreversibly withdraw from division, expression of cell cycle proteins is not completely silenced. Thus, cytoplasmic cyclin D1 was detected in mature neurons associated to the CDKIs p21^Cip1^ and p27^Kip1^, suggesting an impairment of its nuclear transport and a possible role in cell cycle withdrawal [14-16]. Indeed, cyclin D1 is downregulated [17], but also becomes predominantly cytoplasmic, in neuronal progenitor cells undergoing terminal differentiation [18]. Similarly, cyclin E expression was identified in the cytoplasm of postmitotic neurons [19,20]. More recently, Thomas Arendt's lab reported that, within the neocortex of the adult mouse, there is constitutive expression of cyclins D, E, A and B; of CDKs 4, 2 and 1; and of their inhibitors p16^INK4a^, p15^INK4b^, p18^INK4c^, p19^INK4d^, p21^Cip1^, p27^Kip1^ and p57^Kip2 ^[21]. Furthermore, CDKs were found to be properly complexed to cyclins and exhibit kinase activity.

These findings have led to speculate that, in the absence of detectable neuronal cell division, there may be additional, cell cycle independent roles for cell cycle regulators in adult neurons. Indeed, there is evidence to suggest that cyclins and CDKs may participate in synaptic plasticity [22,23] and neuronal differentiation [24,25]. Similarly, CDH1 and APC (anaphase-promoting complex), which are found ubiquitously expressed in the nuclei of terminally differentiated neurons [26], and form a complex involved in cellular division at the end of mitosis and G1 through cyclin B degradation, also appear to play a role in regulating axonal growth and patterning in the developing brain [27]. Furthermore, CDK5, a cyclin-dependent kinase whose exact role in the cell cycle, if any, still remains elusive, is highly active in postmitotic neurons and is involved in the coordination of complex neuronal properties including synaptic plasticity, learning and memory (reviewed in [28]).

Thus, the presence of cell division mediators in differentiated neurons where the cell cycle is absent is well documented, and it does not appear to be the consequence of abnormal regulatory events. Rather, it appears as if at least some cell cycle proteins have adapted to life in a non-dividing neuron by learning and taking up additional, cell cycle-independent roles that are presumably crucial to neuronal function. The use of mouse conditional knockout models of these proteins should help us to unveil both the identity and importance of these putative functions.

### Cell cycle abnormalities in differentiated neurons

There is also a substantial body of evidence pointing to a role for neuronal cell cycle proteins in the modulation of stress-induced apoptosis through a mechanism involving the initiation of a cell cycle. For example, rat cerebellar granule neurons plated in culture medium without trophic factors, such as brain-derived neurotrophic factor (BDNF), undergo apoptosis but also present up-regulated expression of both mRNA and protein levels of cyclin D1. Immunostaining confirmed cyclin D1 immunoreactivity prior to cell shrinkage and nuclear condensation. Furthermore, blocking the cell cycle with the CDKs inhibitors ciclopirox, mimosine and olomoucine was sufficient to suppress immunoreactivity and, more importantly, cell death [6].  Herrup *et al.* showed that two mouse neurological mutants, staggerer (sg/sg) and lurcher (+/Lc), that model the absence of trophic support in the brain, present significant numbers of cerebellar granule cells and inferior olive neurons degenerating after elevation of Cyclin D and proliferating cell nuclear antigen (PCNA) levels and bromodeoxyuridine (BrdU) incorporation [1]. RNA alphavirus Sindbis-driven expression of p16^INK4a^, p21^Cip1^ and p27^Kip1^, and of dominant negative forms of CDK4 and CDK6, protected rat primary neuronal cultures from apoptosis evoked by withdrawal of nerve growth factor (NGF) [2] and neuronal death as a result of DNA-damaging agents treatment, such as camptothecin, AraC and UV radiation [3]. The CDK inhibitors flavopiridol and olomoucine also protected the neurons from these conditions, suggesting that these cell cycle elements might mediate death signalling as a result of DNA-damaging environments [4]. Kruman *et al.* hypothesized that cell cycle reentry is a critical component of the DNA damage response in postmitotic neurons. Suppression of ataxia telangiectasia mutated (ATM), a component of DNA damage-induced checkpoint, by caffeine and wortmannin, attenuated both cell cycle reentry and apoptosis triggered by the genotoxic compounds etoposide, methotrexate, and homocysteine [7].

Oxidative stress-related cell death has also been associated with apparent cell cycle induction in post-mitotic neurons. Induction of cyclin B prior to the commitment of neurons to both dopamine- and peroxide-triggered apoptosis was reported in primary cultures of post-mitotic sympathetic neurons. Both neuronal death and rise in cyclin B were inhibited by antioxidant treatment [5].

In summary, the evidence available to us suggests that exposure of post-mitotic neurons to a wide range of stress stimuli triggers the expression of cell cycle proteins as part of a well regulated programmed cell death response. The most widely accepted scenario is that, in response to stress signals, neurons can be driven into the cell cycle but their array of cell cycle proteins may not suffice to allow for its completion, leading to a situation in which the cell cannot reverse course or complete division, rendering it non-functional and ready to trigger a programmed cell death response. In other words, neurons may have learned to translate stress signals into an irreversibly damaging incomplete cell cycle from which the cell has no choice but to trigger apoptosis. It is also noteworthy in this context that, despite the well-characterized presence of active apoptotic pathways in both in vitro and animal models of AD, the presence of classic apoptotic pathways in the human AD brain is not universally accepted [29]. Thus, it remains formally possible that the cell cycle-linked cell death response in AD, although well documented, may differ in nature from classic apoptosis pathways.

Additional support for this notion is provided by the demonstration of a direct causality link between overexpression of cell cycle mediators and neuronal death. Kranenburg *et al* showed that artificial elevation of cyclin D1 was sufficient to induce apoptosis and could be inhibited by the CDKI p16^INK4 ^[30]. More recently, McShea et al. used adenoviral-mediated expression of c-myc and mutationally active ras oncogenes to force non-dividing cortical neurons into the cell cycle leading to their death [31]. Transgenic mouse models characterized by conditional expression of the simian virus 40 T antigen oncogene in postmitotic neurons clearly presented a neurodegenerative phenotype, consequence of forced cell cycle activation [32].

Nevertheless, even if cell cycle activation is a *sine qua non* for apoptosis in neurons, we still do not know whether the low constitutive levels of cell cycle proteins in neurons may exist to facilitate a fast response to stress or their presence simply reflects their role in unrelated functions.

### Loss of neuronal cell cycle control in AD

If exposure to stress may trigger an abortive cell cycle in neurons, it is reasonable to ask whether such mechanism may exist in the AD brain, which is exposed to a wide range of stress stimuli. Substantial, although mostly descriptive, evidence suggests that this is indeed the case. Cyclins, CDKs and other cell cycle proteins are expressed in the AD brain [9,33-36]. In addition, Ranganathan *et al. *reported high levels of hyperphosphorylated Rb and observed altered subcellular distribution of E2F-1 to the cytoplasm [37] in brain and spinal cord tissues from Alzheimer's disease (AD). In another study, phosphorylated histone H3, a key component involved in chromosome compaction during cell division, was found increased in the cytoplasm of hippocampal neurons in AD, rather than within the nucleus as in actively dividing cells [38]. Cdk7, an activator of major cyclin-CDK complexes, constantly expressed during the cell cycle and indispensable for cell cycle progression, is also upregulated in susceptible hippocampal neurons of AD patients [39].

Further experiments from the Herrup's lab went further in their approach to the study of the neuronal cell cycle and, using fluorescent in situ hybridization, demonstrated that a significant fraction of the hippocampal pyramidal and basal forebrain neurons in AD have fully or partially replicated four separate genetic loci on three different chromosomes [40]. Mosch *et al.*[41] also quantified the DNA amount of identified cortical neurons in AD and reported a population of cyclin B1-positive tetraploid neurons that had entirely passed through a functional interphase with a complete DNA replication. These experiments are particularly important because, unlike evidence showing the presence of cell cycle markers in neurons, which could be dismissed as epiphenomena of suspect physiological relevance, they demonstrate that the DNA replication machinery is functional and capable of completing S phase in post-mitotic neurons.

Interestingly, CDK inhibitors p16^INK4a^, p15^INK4b^, p18^INK4c^ and p19^INK4d^ have also been found abnormally expressed in the temporal cortex and in pyramidal neurons of the hippocampus of AD patients [42-44]. An increase in the cytoplasmic levels of p27^Kip1^ was also identified in vulnerable neurons from individuals with histopathologically confirmed AD [45]. The signifycance of these findings is not immediately obvious. One could argue that expression of these inhibitors occurs as a defence mechanism against the untimely activation of cell cycle initiators. However, that would run counterintuitive to the notion that initiation of an abortive cell cycle is an adaptive response to stress. Clearly, much of the nature of cell cycle events in neurons, whether in response to stress situations or in basal conditions, is far from being understood.

Interestingly, although DNA replication and entry into S phase can be demonstrated to occur in dying neurons, progression through M phase has never been reported. Although the presence of binucleated neurons has been recently reported [46], no condensed chromosomes, formation of a mitotic spindle-like structure, or cytokinesis have ever been described, consistent with the idea that susceptible neurons may be arrested at the G2/M transition before they die. Therefore, activation of CDK1 at G2 might be a rate-limiting step before neurons undergo apoptosis. Indeed, activated CDK1 can phosphorylate and activate the pro-apoptotic BAD protein [47], thus providing a direct link between the cell cycle apparatus and the cell death machinery in neurons. It is also reasonable to suggest, in our opinion, that neuronal apoptosis at the G2 stage may simply be the result of permanent loss of ability to undergo chromosome segregation and cytokinesis due to a highly specialized cytoskeleton. In other words, cytoskeletal commitment to the plasticity of neuronal shape may come at the expense of its inability to dismantle dendrite and axonal structures to commit to mitotic spindle formation and cytokinesis. Indeed, the microtubule associated protein tau, which is phosphorylated during this phase of the cell cycle in a mitotic-competent cell, has also been consistently reported to be abnormally phosphorylated in AD and colocalizes with cell cycle regulators [32,33,45,48-50]. Moreover, tau can be phosphorylated by CDK1 [51] and CDK1-like protein [52,53]. Therefore, abnormally increased levels of tau phosphorylation could be explained in the context of an unsuccessful attempt to modulate G2 neuronal architecture and prepare it for mitosis, leading to programmed cell death.

### Mechanisms of neuronal cell cycle reentry. Lessons from familial AD

Taken together, the available evidence pointing to a role for an abortive cell cycle in neurodegeneration in AD is reasonably strong. Nevertheless, the question remains: what mechanisms do neurons use to enter the cell cycle in the first place in response to a stress signal? If this is an adaptive response, there must be a well-defined molecular pathway that triggers an entry into an apoptotic cell cycle. Although nothing is known in this respect, some clues can be obtained from studies of familial AD (FAD) cases that, perhaps not surprisingly, also display cell cycle abnormalities [54-56].

Mutations in the genes for amyloid precursor protein (APP) and presenilins (PS1, PS2) associated to FAD lead in all cases to aberrant production of Aβ peptides [57], which in turn exacerbate cell cycle-related neuronal death [58-61]. In addition, increased Rb phosphorylation and E2F1 levels are measurable in areas surrounding a subset of Aβ-containing plaques [62]. Interestingly, Copani et al. reported that, unexpectedly, the reparative DNA polymerase β may act as a death signal when erratically expressed by differentiated neurons exposed to Aβ [63]. In short, exposure of post-mitotic neurons to the Aβ levels present in the AD brain may trigger a signalling pathway leading to the initiation of an abortive neuronal cell cycle.

Mutations in Presenilin 1 (PS1) account for the majority of all FAD cases, and one of its functions is precisely the APP γ-secretase-dependent cleavage responsible for Aβ generation. However, PS1 is a multifunctional protein and participates in many other signalling pathways, involving Notch, MEK/ERK, PI3K/Akt, β-catenin and others (reviewed in [64]). Relevant to the present discussion, PS1 is involved in β-catenin proteolysis, coupling its stepwise phosphorylation by PKA and GSK3-β prior to degradation [65-67]. Thus, in the absence of PS1 or in the presence of PS1 FAD mutations, this function is impaired and β-catenin is translocated to the nucleus, leading to hyperproliferation in mitotically competent cells [66-68], and tumorigenesis in peripheral tissue lacking PS1 [69]. Data from our lab points to a β-catenin-dependent aberrant cell cycle reactivation in cultured primary neurons from mice harbouring the knock-in PS1 mutation M146V (PS1 KI^M146V^), as determined by increased BrdU incorporation. This accelerated entry into the cell cycle appeared to be abortive, initiating an apoptotic response. Furthermore, treatment with quercetin, a disruptor of the β-catenin/TCF transcription complex, reduced cyclin D1 levels and reversed the cell cycle/cell death phenotype, consistent with a role for β-catenin in this cell cycle-driven apoptosis [70]. Thus, it is possible that the elevated levels of β-catenin that are present in the PS1 FAD brain accelerate cell cycle entry simply by upregulating cyclin D1 transcription. In further support of this notion, we found that levels of cyclin D1 are elevated in the hippocampus of PS1 FAD patients when compared to sporadic AD patients and non-demented controls (Currais, Hortobagyi and Soriano, unpublished results).

Recently, Repetto et al. demonstrated a critical role for PS1 in the trafficking and turnover of the epidermal growth factor receptor (EGFR), a key signaling receptor tyrosine kinase [71]. As with β-catenin, mutations that enhance EGFR expression can serve as oncogenic signals that promote hyperplasia and neoplastic transformation in human tissues, including skin. EGFR is important for development of the nervous system and maintenance of neural stem cells growth and differentiation. However, excess of EGF induces neuronal death, and strong EGFR immunoreactivity has been detected in neurites surrounding neuritic plaques in AD. Thus, the authors hypothesize that activation of EGFR and β-catenin pathways by the loss of PS1 can mutually reinforce each other and may contribute to neurodegeneration and aberrant cell cycle re-entry by stabilizing both EGFR and β-catenin while simultaneously driving Aβ42 deposition (discussed in [71]).

**Figure 2.**
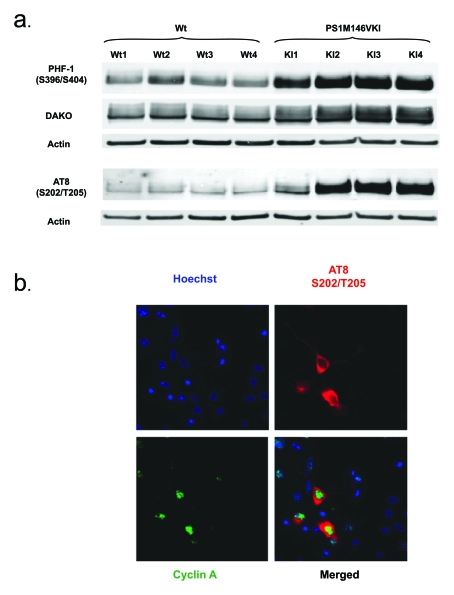
(**a**) Tau accumulates and is hyperphosphorylated at S202/T205 and S396/S404 in primary neurons from PS1 M146V mice compared to wild-type controls. Shown is a Western blot analysis of Triton X-100 soluble lysates. Antibodies used were AT8 (phosphorylated S202/T205), PHF-1 (phosphorylated S396/S404) and DAKO (total tau); (**b**) Tau phosphorylation at S202/T205 is detectable exclusively in neurons expressing cyclin A, highlighting the importance of tau phosphorylation dynamics in the neuronal cell cycle.

Interestingly, and consistent with the notion that a highly specialized cytoskeleton may be the origin of cell cycle-driven apoptosis by simply preventing a cycling neuron from undergoing chromosome segregation and cytokinesis, we have found profound abnormalities in tau homeostasis in our PS1 FAD mouse model. Specifically, tau is hyperphosphorylated in mitotic epitopes in these mice (Figure 2a) and, perhaps more importantly, nuclear expression of cyclin A appears to correlate with the tau phosphorylation at S202/T205 (Figure 2b).

In summary, although the molecular events in a neuron converting a stress stimulus into a signal to enter an abortive cell cycle remain unknown, results from experiments using PS1 FAD models point to the accidental triggering of oncogenic pathways (i.e. aberrant expression of cyclin D1 and EGFR). In that context, tau hyperphosphorylation could be interpreted as a by-product of the attempt by the affected neuron to achieve a mitosis-ready configuration. If this is representative of what occurs in the more widespread non-familial AD cases, we would favour the hypothesis that, rather than an abortive cell cycle being an early event in a regulated cell death response to stress, upregulation of cell cycle proteins in the AD brain may simply reflect the activation of oncogenic pathways that cannot be translated into cell division because of impaired cytoskeletal dynamics, rendering the cell dysfunctional and ready to be eliminated by apoptosis. In further support of this notion, work from the Smith lab has shown that forcing post-mitotic neurons to re-enter the cell cycle through the expression of MYC results in tau changes similar to those seen in AD neurons. More importantly, MYC expression in forebrain neurons of a transgenic model results in cell death and cognitive deficits [31,72]

### Concluding remarks

After differentiation, neurons become post-mitotic, acquiring a structural and functional plasticity at the apparent expense of a permanent exit from the cell cycle. Therefore, the expression of cell cycle markers in the adult brain has always been a subject of controversial debate. Clearly, although neurons are terminally differentiated cells, they do express a wide range of cell cycle proteins and are known to be capable of replicating their DNA, although no cases of a neuronal cell division have ever been reported. This, together with the finding that the expression of cell cycle proteins is necessary to execute apoptosis in response to certain stress signals, has led to the proposition that a neuronal cell cycle does exist and is part of a well-regulated response to stress signals. Whether this interpretation is correct will probably depend on the nature of the initial signal triggering a neuron into the cell cycle in the first place. The fact that cell cycle proteins in neurons are capable of performing non-cell cycle functions and that, at least in PS1-associated FAD, oncogenic signals are readily generated, argue, in our opinion, for a neuronal cell cycle being no different from other oncogenic signals in proliferative cells. The reason for the absence of neuronal division and, indeed, tumors of neuronal origin, would simply reflect the impossibility of a fully mature neuronal cytoskeleton to revert to a mitosis-ready configuration. Clearly, more research is needed before we can begin to understand the physiological and pathogenic implications of a neuronal cell cycle.
